# Upregulation of BUB1B, CCNB1, CDC7, CDC20, and MCM3 in Tumor Tissues Predicted Worse Overall Survival and Disease-Free Survival in Hepatocellular Carcinoma Patients

**DOI:** 10.1155/2018/7897346

**Published:** 2018-09-30

**Authors:** Liping Zhuang, Zongguo Yang, Zhiqiang Meng

**Affiliations:** ^1^Department of Integrative Oncology, Fudan University Shanghai Cancer Center and Department of Oncology, Shanghai Medical College, Fudan University, Shanghai 200032, China; ^2^Department of Integrative Medicine, Shanghai Public Health Clinical Center, Fudan University, Shanghai 201508, China

## Abstract

**Objective:**

To evaluate the association between upregulated differentially expressed genes (DEGs) and the outcomes of patients with hepatocellular carcinoma (HCC).

**Methods:**

Using Gene Expression Omnibus (GEO) datasets including GSE45436, GSE55092, GSE60502, GSE84402, and GSE17548, we detected upregulated DEGs in tumors. KEGG, GO, and Reactome enrichment analysis of the DEGs was conducted to clarify their function. The impact of the upregulated DEGs on patients' survival was analyzed based on TCGA profile.

**Results:**

161 shared upregulated DEGs were identified among GSE45436, GSE55092, GSE60502, and GSE84402 profiles. Cell cycle was the shared pathway/biological process in the gene sets investigation among databases of KEGG, GO, and Reactome. After being validated in GSE17548, 13 genes including BUB1B, CCNA2, CCNB1, CCNE2, CDC20, CDC6, CDC7, CDK1, CDK4, CDKN2A, CHEK1, MAD2L1, and MCM3 in cell cycle pathway were shared in the three databases for enrichment. The expression of BUB1B, CCNB1, CDC7, CDC20, and MCM3 was upregulated in HCC tissues when compared with adjacent normal tissues in 6.67%, 7.5%, 8.06%, 5.56%, and 9.72% of HCC patients, respectively. Overexpression of BUB1B, CCNB1, CDC7, CDC20, and MCM3 in HCC tissues accounted for poorer overall survival (OS) and disease-free survival (DFS) in HCC patients (all log rank* P* < 0.05). BUB1B, CCNB1, CDC7, CDC20, and MCM3 were all overexpressed in HCC patients with neoplasm histologic grade G3-4 compared to those with G1-2 (all* P* < 0.05). BUB1B, CCNB1, and CDC20 were significantly upregulated in HCC patients with vascular invasion (all* P* < 0.05). Additionally, levels of BUB1B, CCNB1, CDC7, and CDC20 were significantly higher in HCC patients deceased, recurred, or progressed (all* P* < 0.05).

**Conclusion:**

Correlated with advanced histologic grade and/or vascular invasion, upregulation of BUB1B, CCNB1, CDC7, CDC20, and MCM3 in HCC tissues predicted worse OS and DFS in HCC patients. These genes could be novel therapeutic targets for HCC treatment.

## 1. Introduction

Hepatocellular carcinoma (HCC) is the fifth most common cancer and the second most common cause of cancer-related deaths [1–3]. In the past two decades, a marked increase in HCC-related annual death rates was observed [2–4]. In addition, the incidence of HCC will continue to rise until 2030 based on a SEER registry projects study [5]. Precise estimation of prognosis plays a critical role in treatment decision in HCC patients. Finding novel biomarkers for predicting HCC prognosis and to reveal HCC target for treatment is urgently needed.

Biomarkers in tumor tissues represent a direct and cost-effective aid in the clinical management of HCC patients, particularly in areas of monitoring disease prognosis and therapeutic target selection. Recently, big data bioinformatics of molecular targets and networks have increasingly gained attention [6, 7], particularly due to the introduction of large scale molecular analysis platforms [8]; human genomes resources of cancers including HCC are publicly available. This tremendous amount of molecular data provides a rich source to better understand the molecular basis of HCC and to identify novel genomic targets for therapeutic intervention. Over the past two decades, advances in high-throughput technologies in biomedical research have led to a dramatic increase in the accessibility of molecular insights at multiple biological levels in HCC [9].

Our study analyzed DEGs between tumor tissues and nontumor tissues in HCC patients based on GEO profiles. Subsequently, the upregulated DEGs were enriched in KEGG, GO, and Reactome, validated in GSE17548 which compared DEGs between HCC tumors and cirrhosis, and evaluated for analysis of HCC outcomes and clinicopathological features. We hope our results could provide useful insights into the potential biomarker candidates and the pathogenesis and progression of HCC patients.

## 2. Materials and Methods

### 2.1. Source of Data

The gene expression profiles of GSE45436, GSE55092, GSE60502, GSE84402, and GSE17548 were downloaded from GEO (https://www.ncbi.nlm.nih.gov/geo/). GSE45436 is composed of GSE45267, GSE45434, and GSE45435. Tumor samples and microarray processing of GSE55092, GSE60502, GSE84402, and GSE17548 were reported by Melis M [10], Wang YH [11], Wang H [12], and Yildiz G [13], respectively.

### 2.2. Identification of Upregulated DEGs in HCC

The gene expression data was processed using the RMA algorithm. To investigate DEGs in transcriptome between tumor tissues and adjacent normal tissues in HCC patients, Affy, AffyPLM, and Limma packages were used for quality assessment and identifying DEGs of tumor and adjacent normal samples in each GEO profile based on the microarray platform. The criteria for selection of DEGs were set as |log_2_⁡FC|> 1 and adjusted* P* value < 0.05. To identify upregulated DEGs, log_2_⁡FC> 1 and adjusted* P* value < 0.05 were set. To identify shared upregulated DEGs among GSE45436, GSE55092, GSE60502, and GSE84402, and to validate the common upregulated genes in GSE17548 which compared DEGs between tumor and cirrhosis tissues, E Chart online service (http://www.ehbio.com/ImageGP/index.php/Home/Index/index.html) for Venn diagram was used.

### 2.3. Functional Enrichment Analysis

KEGG, GO, and Reactome enrichment analysis of upregulated DEGs was conducted using Gene Set Enrichment Analysis (GSEA). To investigate gene sets, upregulated DEGs were uploaded to Molecular Signatures Database in GSEA. A false discovery rate* q-*value cut-off of <0.05 was set as the screening condition. Top 10 KEGG pathways, GO biological process, and Reactome enrichment were presented.

### 2.4. Identification of Candidate Biomarkers for HCC Survival And Clinicopathological Features

To identify potential candidate biomarkers for predicting the overall survival (OS) and disease-free survival (DFS) of HCC patients, Liver Hepatocellular Carcinoma (TCGA, Provisional) database in cBioPortal for cancer genomics web service was used [14, 15]. A z-score threshold ± 2.0 of mRNA expression was selected in genomic profiles and 373 cases with sequenced tumors were conducted for survival analysis. mRNA expression levels calculated by log_2_ were compared based on clinical attribute in HCC patients. To evaluate associations between candidate biomarkers and clinicopathological features in HCC patients, gene data with z scores and clinical data of HCC patients in Liver Hepatocellular Carcinoma (TCGA, Provisional) database were downloaded from cBioPortal and matched with VLOOKUP index in EXCEL.

### 2.5. Statistical Analysis

Differences of gene expression between the individual groups were analyzed using Student's* t*-test or Mann–Whitney* U*-test. PASW Statistics software version 23.0 from SPSS Inc. (Chicago, IL, USA) was used. A two-tailed P<0.05 were considered significant for all tests.

## 3. Results

### 3.1. Screening of Upregulated DEGs

**Figure 1:**
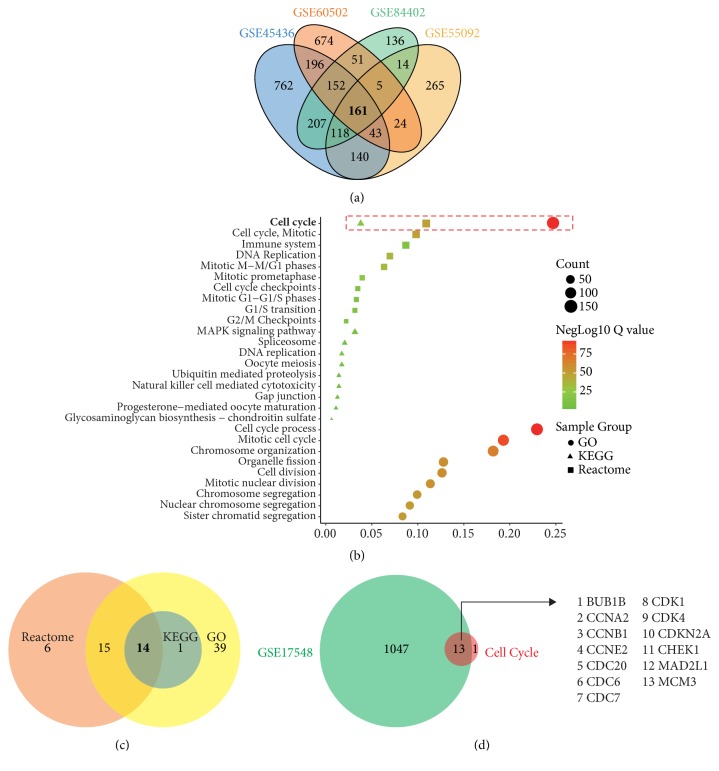
Shared upregulated differential expressed genes (DEGs) of GSE45436, GSE55092, GSE60502, and GSE84402 (a), KEGG, GO, and Reactome enrichment of shared genes from GEO profiles (b), common genes of upregulated DEGs enriched in cell cycle, and (c) validated upregulated DEGs with GSE17548 which compared tumor and cirrhosis tissues in HCC (d).

Totally, overexpression of 1779, 770, 1306, and 844 genes was identified in GSE45436, GSE55092, GSE60502, and GSE84402 profiles, respectively. 161 shared genes were identified among these four GEO profiles using Venn diagram performance (Figure 1(a) and Supplementary Table 1).

### 3.2. Function Analysis of the Upregulated DEGs

To clarify function of the upregulated genes, KEGG pathway, GO biological process, and Reactome gene sets were used for enrichment. We presented top ten pathways/biological processes in our research. As shown in Figure 1(b), cell cycle was the shared pathway/biological process in KEGG, GO, and Reactome (Figure 1(b)). In addition, 15, 69, and 39 genes related cell cycle were enriched in KEGG pathways, GO biological process, and Reactome gene sets, respectively (Figure 1(c)). Subsequently, we conducted Venn diagram and found that 14 genes in cell cycle pathway were shared in the three databases for enrichment (Figure 1(c)). Subsequently, we validated the 14 genes above in GSE17548 profile, which compared DEGs between tumor and cirrhosis tissues in HCC, and 13 genes (BUB1B, CCNA2, CCNB1, CCNE2, CDC20, CDC6, CDC7, CDK1, CDK4, CDKN2A, CHEK1, MAD2L1, and MCM3) were identified finally.

### 3.3. Upregulated Expression of BUB1B, CCNB1, CDC7, CDC20, and MCM3 Predicted Worse Survival in HCC Patients

**Figure 2:**
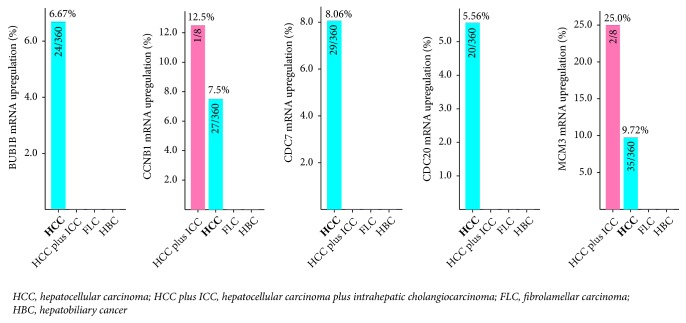
Upregulation frequency of BUB1B, CCNB1, CDC7, CDC20, and MCM3 in different liver cancer types.

**Figure 3:**
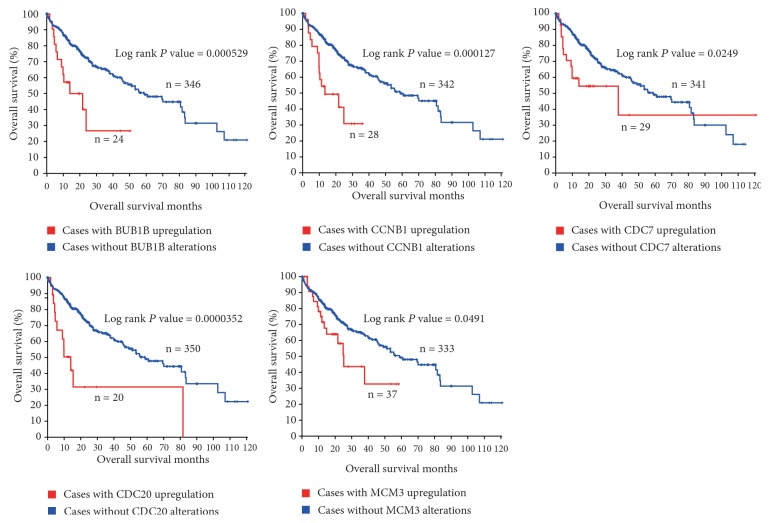
Overall survival of HCC patients grouped by BUB1B, CCNB1, CDC7, CDC20, and MCM3 alterations.

**Figure 4:**
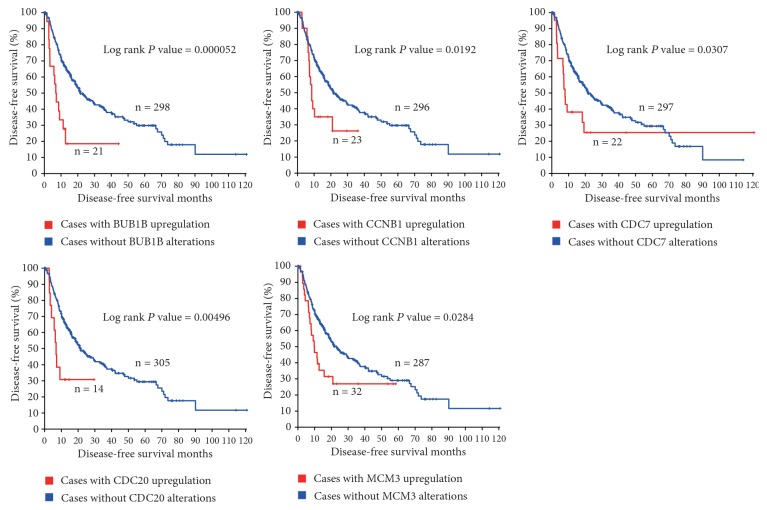
Disease-free survival of HCC patients grouped by BUB1B, CCNB1, CDC7, CDC20, and MCM3 alterations.

Using Liver Hepatocellular Carcinoma (TCGA, Provisional) database in cBioPortal for cancer genomics web service, we included the 13 enriched genes (BUB1B, CCNA2, CCNB1, CCNE2, CDC20, CDC6, CDC7, CDK1, CDK4, CDKN2A, CHEK1, MAD2L1, and MCM3) for identifying potential candidate biomarkers for OS and DFS in HCC patients. As shown in Figure 2, BUB1B, CCNB1, CDC7, CDC20, and MCM3 were upregulated in HCC tissues in 6.67%, 7.5%, 8.06%, 5.56%, and 9.72% of HCC patients, respectively. Additionally, overexpression of BUB1B, CCNB1, CDC7, CDC20, and MCM3 in HCC tissues accounted for poorer OS in HCC patients (Log rank* P* = 0.000529, 0.000127, 0.0249, 0.0000352, and 0.0491, respectively, Figure 3 and Supplementary Table 2). Upregulated BUB1B, CCNB1, CDC7, CDC20, and MCM3 in HCC tumor tissues also contributed to worse DFS in HCC patients (Log rank* P *= 0.000052, 0.0192, 0.0307, 0.00496, and 0.0284, respectively, Figure 4 and Supplementary Table 3).

### 3.4. Links between BUB1B, CCNB1, CDC7, CDC20, and MCM3 and Clinicopathological Features in HCC Patients

**Figure 5:**
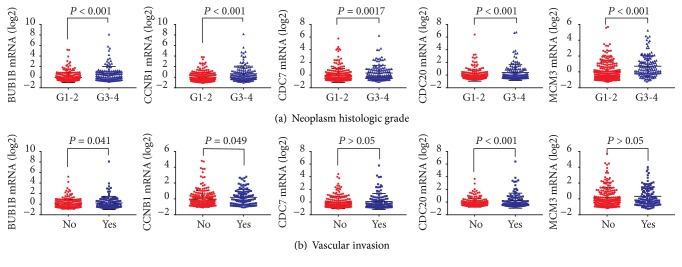
BUB1B, CCNB1, CDC7, CDC20, and MCM3 expression of HCC patients based on neoplasm histologic grade (a) and vascular invasion (b).

**Figure 6:**
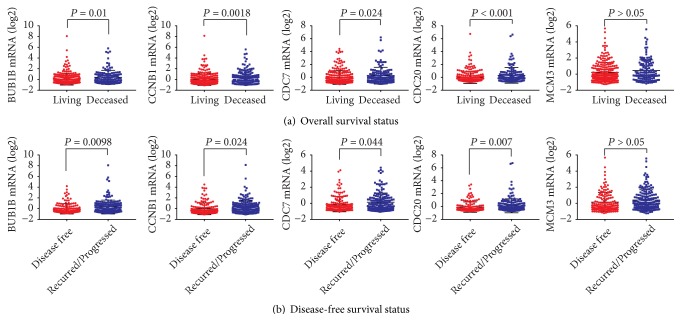
BUB1B, CCNB1, CDC7, CDC20, and MCM3 expression of HCC patients based on overall survival status (a) and disease-free survival status (b).

As shown in Figure 5, BUB1B, CCNB1, CDC7, CDC20, and MCM3 were significantly increased in HCC patients with neoplasm histologic grade G3-4 compared to those with G1-2 (all* P* < 0.05, Figure 5(a)). In addition, HCC patients with vascular invasion had higher BUB1B, CCNB1, and CDC20 levels than those without vascular invasion (all* P* < 0.05, Figure 5(b)). As shown in Figure 6, BUB1B, CCNB1, CDC7, and CDC20 were significantly overexpressed in deceased, recurred, or progressed HCC patients (all* P* < 0.05, Figure 6).

## 4. Discussion

It has been well studied that cell cycle regulators are strongly implicated in progression of cancer development [16]. Disruption of the cell cycle pathway has previously been associated with development of several kinds of cancers, including HCC [17]. Although recent progress has enabled improved diagnosis and management of HCC, its prognosis remains dismal. Identification of favorable prognostic biomarkers linked to HCC outcomes is a critical step for developing an efficient treatment.

To find candidate biomarkers for HCC prognosis, we identified upregulated genes in HCC tumor tissues based on four GEO profiles. In our study, we found that the most frequently upregulated genes in HCC tumor tissues were enriched in cell cycle pathway. BUB1B, CCNB1, CDC7, CDC20, and MCM3 were identified as potential predictors for OS and DFS of HCC patients. In addition, overexpression of BUB1B, CCNB1, CDC7, CDC20, and MCM3 also contributed to advanced histologic grade and/or vascular invasion. Hence, we assumed that BUB1B, CCNB1, CDC7, CDC20, and MCM3 should be candidate biomarkers for HCC development and promising treatment targets.

As a checkpoint for proper chromosome segregation and preventing separation of the duplicated chromosomes in normal cells, the role of BUB1B (encoding BUBR1) in cancer cells is still controversial. Low expression of BUB1B contributes to poor survival and metastasis in human colon adenocarcinomas [18] and lung cancer [19], while overexpression of BUB1B is related to progression and recurrence of gastric cancer [20], bladder cancer [21], HCC [22], and many other cancers [23–25]. Encoded by BUB1B, high expression of the BUBR1 was correlated with larger tumor size, higher histological grade, advanced pathological stage, and poor survival in HCC patients [22], which is in line with our results. CCNB1 (also known as CyclinB1) serves as a vital regulator of cell cycle, which is significantly overexpressed in various cancer types. Previous studies revealed that CCNB1 promotes cell proliferation, tumor growth, and cancer recurrence and relates to progression and survival in various cancers [26–30]. As cell cycle regulating kinases, CDC7 has been shown to be necessary to initiate the S phase and CDC20 is an essential cell-cycle regulator required for the completion of mitosis. Overexpression of CDC7 in malignant tumors correlates with tumor differentiation [31] and poor prognosis in patients with B-cell lymphoma [32]. CDC20 may function as an oncoprotein to promote the development and progression of human cancers. CDC20 has been reported to be significantly elevated in tumor tissues with poor differentiation and has been linked to poor prognosis in pancreatic cancer [33], lung cancer [34], bladder cancer [35], colon cancer [36], oral squamous cell carcinomas [37], and breast cancer [38]. Inhibitors of CDC7 [39–41] and CDC20 [42, 43] kinases would be promising candidates for novel classes of cancer drugs. MCM3 is a novel proliferation marker and is useful to determine the clinical behavior and prognosis in several cancers [44]. Previous studies showed that high MCM3 expression is an independent biomarker for poor prognosis of malignant melanoma [45] and epithelial ovarian cancer [46]. Unfortunately, few studies of CCNB1, CDC7, CDC20, and MCM3 were published for evaluating correlations to HCC clinicopathological features and outcomes. According to our results, we considered the aforementioned genes to be predictive biomarkers for survival of HCC patients and to be therapeutic targets.

Our study should be considered in the context of its limitations. First, BUB1B, CCNB1, CDC7, CDC20, and MCM3 genes were examined in transcription levels, not in protein levels. Second, no mechanisms of these genes were conducted, such as gene silencing approaches. We suggested future studies focused on the associations between these genes and HCC progression and development, both basically and clinically.

In summary, we concluded that upregulation of BUB1B, CCNB1, CDC7, CDC20, and MCM3 in HCC tissues correlated to poor histological grade and/or more risk of vascular invasion. Overexpression of these genes could predict worse OS and DFS in HCC patients. Considering previous reports, we hypothesized that BUB1B, CCNB1, CDC7, CDC20, and MCM3 should be novel prognostic biomarkers and promising therapeutic targets for HCC patients.

## Data Availability

All the data in this study are available from GEO database (https://www.ncbi.nlm.nih.gov/geo/) and TCGA database from cBioPortal for Cancer Genomics (http://www.cbioportal.org/).

## Abbreviations

HCC: Hepatocellular carcinoma
DEG: Differential expressed genes
GEO: Gene Expression Omnibus
GSEA: Gene Set Enrichment Analysis
BUB1B: Budding uninhibited by benzimidazoles 1 homolog beta
CCNB1: Cyclin B1
CDC7: Cell division cycle 7 homologue
CDC20: Cell division cycle 20 homologue
MCM3: Minichromosome maintenance protein 3
OS: Overall survival
DFS: Disease-free survival.

## Supplementary Materials

Supplementary Materials Supplementary Table 1: 161 upregulated differential expressed genes in GSE45436, GSE55092, GSE60502, and GSE84402 profiles. Supplementary Table 2: overall survival of HCC patients based on BUB1B, CCNB1, CDC7, CDC20, and MCM3 alterations. Supplementary Table 3: disease-free survival of HCC patients based on BUB1B, CCNB1, CDC7, CDC20, MCM2, and MCM3 alterations.
