# Supplementary material for: Upregulation of BUB1B, CCNB1, CDC7, CDC20, and MCM3 in Tumor Tissues Predicted Worse Overall Survival and Disease-Free Survival in Hepatocellular Carcinoma Patients
Source: Biomed Res Int. 2018 Sep 30;2018:7897346. doi: 10.1155/2018/7897346 (PMC6186344; doi:10.1155/2018/7897346)
Supplement: Supplementary Materials — Supplementary Table 1: 161 upregulated differential expressed genes in GSE45436, GSE55092, GSE60502, and GSE84402 profiles. Supplementary Table 2: overall survival of HCC patients based on BUB1B, CCNB1, CDC7, CDC20, and MCM3 alterations. Supplementary Table 3: disease-free survival of HCC patients based on BUB1B, CCNB1, CDC7, CDC20, MCM2, and MCM3 alterations. [file 7897346.f1.docx]

**Supplementary Table 1** 161 upregulated differential expressed genes in GSE45436, GSE55092, GSE60502 and GSE84402 profiles

| 1 ACSL4 | 41 EFNA4 | 81 LPCAT1 | 121 RACGAP1 |
| --- | --- | --- | --- |
| 2 AKR1C3 | 42 ENAH | 82 LPL | 122 RAD51AP1 |
| 3 ASPM | 43 ERV3-2 | 83 MAD2L1 | 123 RCN2 |
| 4 ASRGL1 | 44 ESM1 | 84 MAGEA12 | 124 RFC4 |
| 5 ATAD2 | 45 EZH2 | 85 MAP4K4 | 125 ROBO1 |
| 6 AURKA | 46 FABP5 | 86 MCM10 | 126 RPS6KC1 |
| 7 BUB1B | 47 FAM50A | 87 MCM2 | 127 RRAGD |
| 8 C5orf30 | 48 FANCI | 88 MCM3 | 128 RRM2 |
| 9 CAP2 | 49 FEN1 | 89 MEA1 | 129 S100P |
| 10 CCL20 | 50 FLAD1 | 90 MELK | 130 SAC3D1 |
| 11 CCNA2 | 51 FOXM1 | 91 MKI67 | 131 SERPINI1 |
| 12 CCNB1 | 52 G6PD | 92 MMP12 | 132 SGCE |
| 13 CCNE2 | 53 GINS1 | 93 MMP9 | 133 SLC26A2 |
| 14 CCT6A | 54 GLA | 94 MSH2 | 134 SLC38A6 |
| 15 CDC20 | 55 GMNN | 95 MUC13 | 135 SMC4 |
| 16 CDC6 | 56 GNPAT | 96 NAA40 | 136 SMYD3 |
| 17 CDC7 | 57 GPC3 | 97 NCAPG | 137 SPAG4 |
| 18 CDCA3 | 58 GPSM2 | 98 NCAPG2 | 138 SPATS2 |
| 19 CDK1 | 59 GSTA4 | 99 NDC80 | 139 SPINK1 |
| 20 CDK4 | 60 GTF2IRD1 | 100 NEK2 | 140 SQLE |
| 21 CDKN2A | 61 H2AFZ | 101 NEU1 | 141 STIL |
| 22 CDKN3 | 62 HAUS3 | 102 NME1 | 142 STK39 |
| 23 CENPE | 63 HELLS | 103 NQO1 | 143 SUCO |
| 24 CENPF | 64 HIST2H2BE | 104 NRAS | 144 SULT1C2 |
| 25 CENPM | 65 HJURP | 105 NRCAM | 145 TARBP1 |
| 26 CENPU | 66 HMMR | 106 NUSAP1 | 146 TKT |
| 27 CEP55 | 67 HN1 | 107 OIP5 | 147 TMEM38B |
| 28 CHEK1 | 68 HSPB1 | 108 OLFML2B | 148 TOP2A |
| 29 CKAP4 | 69 IGF2BP3 | 109 PAFAH1B3 | 149 TP53I3 |
| 30 CKS2 | 70 ITGA6 | 110 PBK | 150 TPX2 |
| 31 CLGN | 71 KIAA0101 | 111 PDZK1 | 151 TRIM24 |
| 32 COG2 | 72 KIF11 | 112 PEG10 | 152 TRIP13 |
| 33 COL15A1 | 73 KIF14 | 113 PODXL | 153 TTK |
| 34 CTC-338M12.4 | 74 KIF18B | 114 POLQ | 154 TUBG1 |
| 35 DDX39A | 75 KIF20A | 115 PRC1 | 155 UBAP2L |
| 36 DKK1 | 76 KIF4A | 116 PRCC | 156 UBE2C |
| 37 DLGAP5 | 77 KNTC1 | 117 PRIM1 | 157 WASF1 |
| 38 DTL | 78 KPNA2 | 118 PSMD4 | 158 XPOT |
| 39 E2F8 | 79 LAMC1 | 119 PSPH | 159 ZNF43 |
| 40 ECT2 | 80 LOC81691 | 120 PSRC1 | 160 ZNF468 |
|  |  |  | 161 ZWINT |

**Supplementary Table 2** Overall survival of HCC patients based on BUB1B, CCNB1, CDC7, CDC20 and MCM3 alterations

| Gene | Group | Total cases, n | Deceased cases, n | Median survival months |
| --- | --- | --- | --- | --- |
| BUB1B | Upregulation | 24 | 12 | 13.96 |
|  | No alterations | 346 | 118 | 58.84 |
| CCNB1 | Upregulation | 28 | 14 | 13.47 |
|  | No alterations | 342 | 116 | 58.84 |
| CDC7 | Upregulation | 29 | 13 | 37.75 |
|  | No alterations | 341 | 117 | 58.84 |
| CDC20 | Upregulation | 20 | 12 | 13.96 |
|  | No alterations | 350 | 118 | 58.84 |
| MCM3 | Upregulation | 37 | 15 | 25.23 |
|  | No alterations | 333 | 115 | 58.84 |

**Supplementary Table 3** Disease-free survival of HCC patients based on BUB1B, CCNB1, CDC7, CDC20, MCM2 and MCM3 alterations

| Gene | Group | Total cases, n | Relapsed/Progressed cases, n | Median survival months |
| --- | --- | --- | --- | --- |
| BUB1B | Upregulation | 21 | 14 | 6.87 |
|  | No alterations | 298 | 160 | 21.62 |
| CCNB1 | Upregulation | 23 | 14 | 8.54 |
|  | No alterations | 296 | 160 | 21.55 |
| CDC7 | Upregulation | 22 | 15 | 7.88 |
|  | No alterations | 297 | 159 | 21.62 |
| CDC20 | Upregulation | 14 | 9 | 6.87 |
|  | No alterations | 305 | 165 | 21.16 |
| MCM3 | Upregulation | 32 | 20 | 9.76 |
|  | No alterations | 287 | 154 | 23.03 |
